# Utilising unsupervised machine learning to predict outbreaks of respiratory tract infections in acute Irish hospitals (2016-2021)

**DOI:** 10.1016/j.puhip.2026.100748

**Published:** 2026-02-07

**Authors:** Doaa Amin, Akke Vellinga

**Affiliations:** a CARA Network, School of Public Health, Physiotherapy and Sports Science, University College Dublin, Room A101, Health Sciences Building, Belfield, Dublin 4, Ireland; b CARA Network, School of Public Health, Physiotherapy and Sports Science, University College Dublin, Woodview House, Belfield, Dublin 4, Ireland

**Keywords:** Machine Learning (ML), Unsupervised machine learning, K-modes clustering, Hierarchical clustering, Respiratory Tract infections (RTIs), COVID-19

## Abstract

**Objectives:**

To apply unsupervised machine learning (ML) to predict outbreaks of respiratory tract infections (RTIs) in acute Irish hospitals (2016-2021).

**Study design:**

A retrospective study.

**Methods:**

RTIs data was extracted from Irish hospital inpatient enquiry (HIPE). Three k-modes clustering models were developed, whose resulting clusters were compared via graphical visualisation of main RTIs to choose the model which captured the outbreaks best. To understand the individual RTIs behind the outbreaks, further exploration was carried out.

**Results:**

Nearly half a million patients (491,099) were admitted to 55 acute Irish hospitals with an RTI. Model 2, including 212 diagnostic groups according to hierarchical clustering, was able to capture all outbreaks. Further analysis resulted in five diagnostic codes that contributed with two thirds of all RTI hospitalisations throughout the six years (acute lower RTI (28.24%), pneumonia (20.76%), chronic obstructive pulmonary disease with acute lower RTI (7.52%), COVID-19 (2020-2021) (5.13%), and acute upper RTI (4.37%)).

**Conclusion:**

Unsupervised ML (K-modes clustering) can be useful in predicting RTIs outbreaks in acute Irish hospitals. Further analysis identified five RTI diagnostic codes that contributed most to outbreaks, which if monitored, may alert hospitals of potential RTI outbreaks.

**What this study adds**

• K-modes clustering algorithm can be useful in predicting RTI outbreaks in acute hospitals in Ireland,
• Grouping diagnostic codes with hierarchical clustering can eventually lead to better predictive results for the RTI outbreaks,
• Five diagnostic codes (i.e., infections) were responsible for two thirds of all hospitalisations with an RTI in acute Irish hospitals (2016-21): acute lower RTI (28.24%), pneumonia (20.76%), chronic obstructive pulmonary disease with acute lower RTI (7.52%), COVID-19 (2020-2021) (5.13%), and acute upper RTI (4.37%).

**Implications for policy and practice**

• The early identification of potential RTI outbreaks can inform national-level policies in Ireland for outbreak preparedness and responsiveness,
• Understanding the individual diagnostic codes that contribute the most to the outbreaks may help in the introduction of early warning systems in hospitals for potential outbreaks and in planning vaccination campaigns.

## 1 Introduction

RTIs are one of the main causes of mortality and morbidity worldwide, with the older and infant population at the highest risk [1,2]. Furthermore, there is an economic burden due to RTIs, reflected in the increased utilisation of healthcare services and resources, and the decreased productivity due to illness [3]. In 2019, RTI was the cause of death for one in every seven children under the age of 5 and in the top 10 of global causes of death and disability-adjusted life years for adults [4,5]. In Ireland, total registered deaths in the under 65 year olds for pneumonia was 0.8% and 2.3% in the over 65 year-olds (2021) [6]. In addition, Covid-19 which is also considered an RTI, resulted in more than 7 million deaths globally and more than 9300 deaths in Ireland [7,8].

Unsupervised machine learning is one of three main categories for machine learning (ML), along with supervised and reinforcement learning [9,10]. Unsupervised ML algorithms are trained on unlabelled data that do not include information about outcomes but are used to explore patterns in data and generate new hypotheses [[11], [12], [13]]. Examples of unsupervised ML are clustering algorithms and dimensionality reduction [14]. In clustering algorithms, the aim is to group data based on a similarity measure or feature [[15], [16], [17]]. Clustering algorithms have different applications in healthcare, such as identifying patients' profiles, using plasma biomarkers to identify older adults at risk of Alzheimer's disease and related dementias identifying subgroups of patients with heart failure using telehealth, and clustering of functional magnetic resonance imaging (fMRI) data [[18], [19], [20], [21]].

An outbreak is defined as: “two or more linked cases of the same illness or the situation where the observed number of cases exceeds the expected number, or a single case of disease caused by a significant pathogen (e.g. diphtheria or viral haemorrhagic fever). Outbreaks may be confined to some of the members of one family or may be more widespread and involve cases either locally, nationally or internationally” [22]. No previous studies were identified on predicting RTIs outbreaks in Ireland, or understanding the burden of individual or combination of RTI presentations in initiating the outbreaks. Thus, this study applied an unsupervised machine learning algorithm to predict outbreaks of RTIs in acute Irish hospitals (2016-2021).

## 2 Methods

A retrospective analysis was carried out on inpatient data in 55 acute hospitals in Ireland. RTI data (2016-2021) was obtained from the hospital inpatient enquiry (HIPE), an information system that contains data of inpatients from acute hospitals in Ireland [23]. The fully anonymised RTI dataset, from 55 acute Irish hospitals (2016-2021), included gender, age group, month and year of admission and discharge, and 30 diagnoses (principal and up to 29 secondary diagnoses). The diagnoses of inpatients are coded according to the international classification of diseases, 10th revision (ICD-10) [24] and these diagnostic codes extracted from HIPE were all for RTIs including Covid-19 [Appendix 1]. For the K-modes clustering analysis carried out in this study, a data subset was created including age group, four diagnoses for every inpatient (a principal and the three subsequent secondary diagnoses) and a “MonthYear” variable representing month and year of admission. In addition, diagnostic codes (i.e., ICD-10 codes) for COVID-19 were unified into one code.

In comparison with other prediction models, the K-modes clustering algorithm, based on Cao initialisation, was chosen for its suitability for categorical data [25,26] [Appendix 2].

To avoid noise (i.e. the inclusion of non-RTI diagnostic codes), the individual diagnostic codes in the RTIs dataset were replaced by diagnostic groups, which were either retrieved from the World Health Organisation (WHO)'s logical categorisation of disease groups or developed by hierarchical clustering. The optimal number of clusters “K” is determined by using the elbow method [27,28] [Appendix 3]. The inputs for a k-modes clustering model were the dataset, the number of clusters, and some hyperparameters such as the initialisation method, which was Cao in our study, and specifying the random state, zero in our study, to ensure reproducibility.

Three K-modes clustering models were developed, with the number of clusters set at (K), determined by the Elbow method, to test which model would capture outbreaks best:

• *Model 1:* allocated diagnostic groups according to the WHO logical diagnostic groups [29].
• *Model 2:* applied an equal number of diagnostic groups to Model 1, that were grouped according to hierarchical clustering (HC) based on the “Ward” method and a penalty for alphabetic similarity was added (based on Jaccard distance) [[30], [31], [32]]. HC was chosen for its suitability for the categorical nature of the diagnostic codes (ICD-10 codes), as well as its ability to discover the hierarchical patterns between the different ICD-10 codes [Appendix 2]. In addition, the number of diagnostic groups (n = 212) were based on an arbitrary cut-off in a dendrogram (i.e. “a two-dimensional diagram representing a tree of relationships, whatever their nature” [33]) [Appendix 4].
• *In addition, a third model (Model 3)*: was developed to test if fewer number of diagnostic groups would improve prediction. As well as in Model 2, the number of diagnostic groups (n = 80) were based on an arbitrary cut-off in a dendrogram [33] [Appendix 4].

Due to the large size of the dataset, its categorical nature and to avoid a dimensionality problem if one-hot encoding was performed on the dataset for, the cost (i.e., dissimilarity measure) of the models and the average distance between centroids of clusters (based on Jaccard distance) were used as performance measures.

Furthermore, the resulting clusters from the three models were compared to a graphical visualisation for groups of RTIs over time. All analyses and graphical visualisations were carried out using Python 3.11.5.

## 3 Results

A total of 491,099 inpatients with an RTI were admitted to 55 acute Irish hospitals (2016-2021). Three k-modes clustering models were developed with three different diagnostic groupings, where in Model 1, 212 logical diagnostic groups were used, according to WHO classification [29]. On the other hand, Model 2 had an equal number of diagnostic groups as in Model 1, 212 groups, however, they were grouped by using hierarchical clustering. Finally, Model 3 had 80 diagnostic groups using hierarchical clustering as well. The Elbow method was applied to find optimal number of clusters for each of the 3 models. The K-modes clustering algorithm was carried out at K = 6 [Appendix 3].

**Fig. 1:**
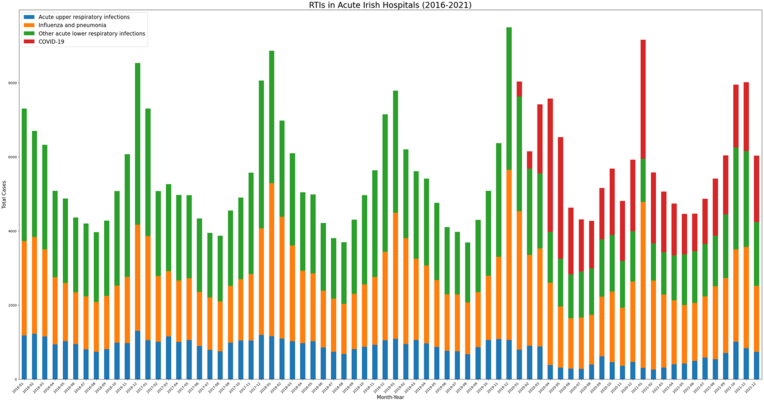
Main RTIs in acute Irish hospitals (2016-2021).

In addition, a graphical visualisation for main groups of RTIs (Acute upper RTI, Influenza and pneumonia, Other lower RTI, and COVID-19 (2020-2021)) was developed and used as a point of comparison between the results of the three models [Fig. 1]. The stacked plot showed annual outbreaks in hospitalisations with an RTI around the winter months (2016-2021). In 2020-21, the slight decrease in hospitalisations with upper RTIs, influenza and pneumonia, and lower RTIs were replaced with COVID-19 hospitalisations, resulting in a similar pattern to previous years (2016-19)

### 3.1 Model 1 with the 212 logical diagnostic groups

**Fig. 2:**
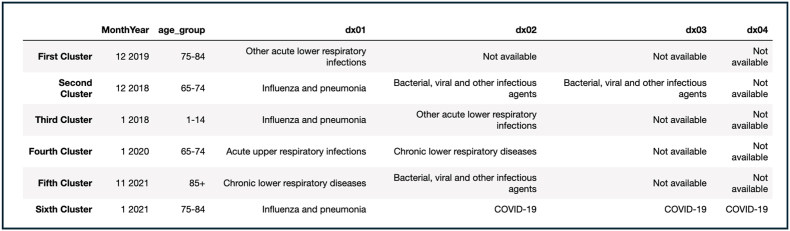
K-modes Clustering (with 212 logical diagnostic groups) – Model 1 (where dx01-04 are the first four diagnoses of an inpatient).

All the resulting clusters took place around winter months (Nov-Jan 2018-2021), with the elderly appearing in most of them, except for the third cluster which included 1-14 year olds (Fig. 2). There were six logical diagnostic groups that appeared in the clusters, such as other acute lower respiratory infections, influenza and pneumonia, bacterial, viral and other infectious agents, acute upper respiratory infections, chronic lower respiratory diseases and COVID-19 (only in the sixth cluster taking place in 2021). The model's total cost (i.e., dissimilarity measure) was 2,018,794 and the average distance between centroids was 0.85. When the MonthYear variable in the different clusters was compared to the outbreaks (i.e., peaks) in Fig. 1, two peaks were incorrectly captured (Dec 2018 (second cluster) and Jan 2020 (fourth cluster)). Model 1 captured 66.7% of the outbreaks throughout the six years.

### 3.2 Model 2 with the 212 diagnostic groups developed by HC

**Fig. 3:**
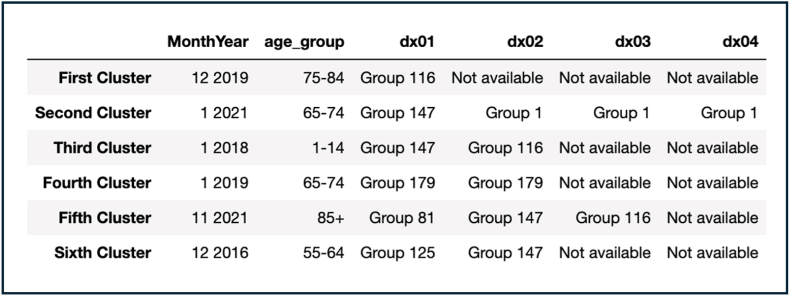
K-modes Clustering (with 212 diagnostic groups by HC) – Model 2.

As well as in Model 1, all the resulting clusters took place in the winter (Nov-Jan 2016, 2018-19, 2021). Age groups were the same as the ones resulting in Model 1, except for the introduction of the age group 55-64 year olds (sixth cluster) (Fig. 3). Six diagnostic groups appeared in the different clusters (i.e., Group 1, Group 81, Group 116, Group 125, Group 147, Group 179). The total cost of the model was 2,064,520, which is higher than Model 1, however the average distance between the centroids was 0.83, which is somewhat lower than Model 1 reflecting a slight improvement in the similarity between centroids. When compared to Fig. 1, the model captured 100% of the outbreaks, and captured more outbreaks than Model 1 such as Jan 2019 and Dec 2016.

To better understand the ability of Model 2 in capturing outbreaks correctly, as well as the individual diagnostic codes that may have initiated the outbreaks, the six groups appearing in the resulting clusters of Model 2 were further explored. There were 11 diagnostic codes relevant to RTIs included in these six groups (J22: acute lower RTI, J18.9: pneumonia, J44.0: chronic obstructive pulmonary disease with acute lower RTI, U07.1: COVID-19 (2020-2021), J06.9: acute upper RTI, J09: influenza due to identified zoonotic or pandemic influenza virus, J04.0: acute laryngitis, J40: bronchitis, B96.0: mycoplasma pneumoniae, B90.0: sequelae of respiratory & unspecified Tuberculosis, and A19.8: Tuberculosis). Of those codes, five were responsible for two thirds (66%) of all presentations with an RTI in acute Irish hospitals throughout the six years (acute lower RTI (28.24%), pneumonia (20.76%), chronic obstructive pulmonary disease with acute lower RTI (7.52%), COVID-19 (2020-2021) (5.13%), and acute upper RTI (4.37%)) [Panel 1].

In Panel 1, the graphs for acute lower RTI, pneumonia, chronic obstructive pulmonary disease with acute lower RTI show that the elderly were more affected (over 65 year olds) and the age has decreased for COVID-19 to include the over 45 year olds. This was different for upper RTIs where the age group influenced the most was the 1-14 year olds. In addition, all these five infections had their outbreaks during the winter months.

To ensure distinct clusters were formed, two-dimensional multiple correspondence analysis (MCA) was carried out. MCA is a technique that is utilised to detect relationships between categorical data and it allows for non-linearity to emerge [34]. The analysis was carried out using the Prince library in Python [35]. The eigenvalues represent the contribution of each dimension to the total inertia, and their sum is 1, which reflects the total information about all the variables in all of the dimensions) [36] (Fig. 5).

**Fig. 4:**
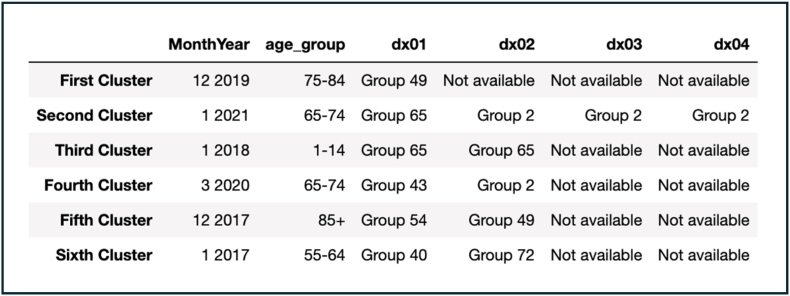
K-modes Clustering (with 80 diagnostic groups by HC) – Model 3.

**Fig. 5:**
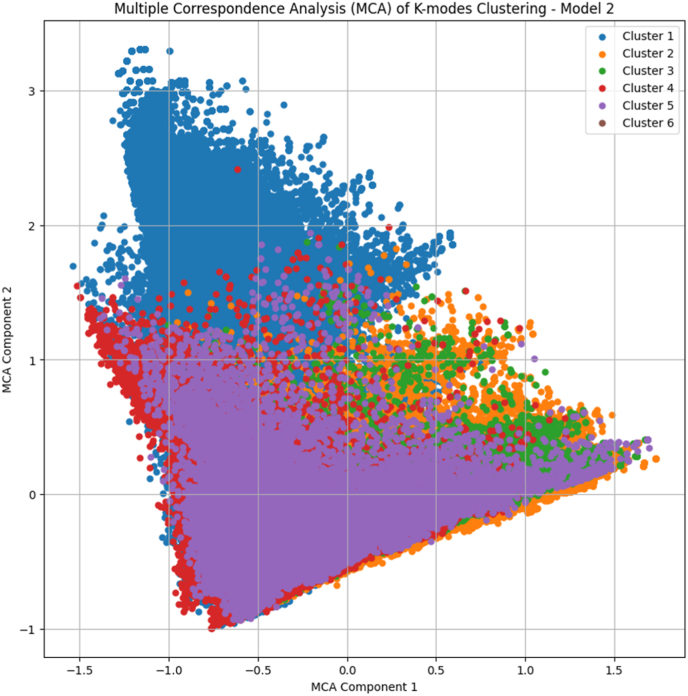
Multiple correspondence analysis (MCA) – Model 2.

The resulting eigenvalues were [0.546 (54.6%), 0.452 (45.2%)] respectively, which summed up to around 99.8% of the total inertia (i.e., variance) explained by the two dimensions (i.e., axes) in Fig. 5. In addition, the proximity of the six clusters in the MCA plot indicated similar patterns across the temporal, demographical and diagnostic variables utilised for clustering (i.e., MonthYear, Age_group, and Diagnosis 1 - 4).

**Panel 1:**
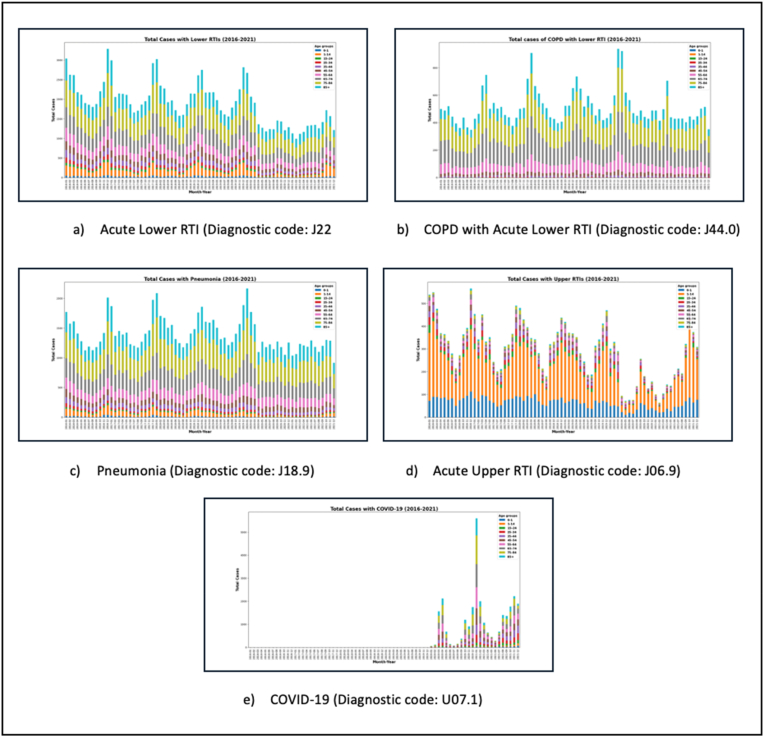
The five main diagnostic codes responsible for two thirds of RTI hospitalisations (2016-2021).

### 3.3 Model 3 with the 80 diagnostic groups developed by HC

Similar to Model 1 &2, most of the resulting clusters were in the winter (Dec-Jan 2017-19, 2021) except for Mar 2020 (Fig. 4). The age groups were more similar to those appearing in Model 2 and there were 7 diagnostic groups appearing in the resulting clusters (Group 2, Group 40, Group 43, Group 49, Group 54, Group 65, Group 72). The Model's cost was 2,052,704, which is higher than Model 1 but lower than Model 2. And the average distance between centroids was 0.88 which is higher than both Models 1 & 2 reflecting less compact and further clusters. More similar to Model 1, this model did not capture all outbreaks correctly (66.7%) and ended up capturing completely wrong peaks, such as Mar 2020. Since this model showed worse performance than Models 1 & 2, therefore no further models were explored.

## 4 Discussion

Almost half a million of inpatients with an RTI were admitted to acute Irish hospitals (2016-2021). An unsupervised ML model, a K-modes clustering model, which included 212 diagnostic groups grouped by HC, was able to predict outbreaks of RTIs best. The resulting clusters showed outbreaks around winter months, with the older adults (more than 55 year olds) and children (1-14 year olds) being the most common. Further exploration of the diagnostic groups in in this model showed that five individual diagnostic codes (J22: acute lower RTI, J18.9: pneumonia, J44.0: chronic obstructive pulmonary disease with acute lower RTI, U07.1: COVID-19 (2020-2021), and J06.9: acute upper RTI) were behind two thirds of all hospitalisations with an RTI throughout the six years, except for COVID-19 only in 2020-21.

Previously, approaches based on traditional statistical methods were used for surveillance purposes, and predicting and/or preparing for outbreaks in RTIs. For example, multivariable analysis was used for outbreak preparation for acute RTIs [37], and Gaussian time series models were used for detecting the signatures of an RTI [38]. However, ML models offered several advantages over those, such as flexibility as well as the ability to use different types of data (i.e., clinicians free text, laboratory data, as well as patients demographics) for different prediction purposes (i.e., diagnosis, or risk of a disease) [39].

To the best of our knowledge, our study is the first to apply an unsupervised ML model, a K-modes clustering model, for predicting outbreaks of RTIs in Ireland. Applying this method had additional advantages, such as its suitability for the categorical nature of the HIPE dataset without the need for any data transformation, as well as its ability to handle the large size of the dataset. This method, if applied in secondary care in Ireland, can be an added value to disease surveillance and prevention efforts. In addition, our finding of the five main contributing diagnostic codes to the outbreaks, may help in the introduction of early warning systems in hospitals for potential outbreaks and in planning vaccination campaigns. Furthermore, early identification of potential RTI outbreaks can inform national-level policies in Ireland for outbreak preparedness and responsiveness.

In Ireland, the outbreaks of RTIs (Model 2) were around the winter months, which was consistent with findings of studies from Belgium, China, Hanoi (Vietnam), and Madrid and Barcelona (Spain) [[40], [41], [42], [43]]. In addition, other studies reported results about infected age groups with an RTI that were more similar to our finding (older adults and elderly (55-85+ year olds) and children (1-14 year olds)) [44,45]. Furthermore, a previous study reported that high numbers of hospitalisations with an RTI were associated with people with chronic conditions, which was in agreement with our finding about COPD being one of the five diagnostic codes responsible for the majority of the hospitalisations with an RTI [46].

A key strength of the K-modes clustering models utilised in this study, is that they can capture the data with high recurring patterns, which enabled the identification of groups of inpatients that follow similar temporal/diagnostic patterns.

One of the limitations of unsupervised ML models is that they do not handle the periodic nature of datasets well [47]. However, in our analysis, the “MonthYear” variable was treated as categorical to overcome any temporal or cyclic ordering in the data. Accordingly, the K-modes clustering models could reflect diagnostic similarities in the Irish electronic health records (i.e., HIPE data).

Future research could explore if our model is suitable to predict outbreaks of RTIs in countries with close healthcare structure to Ireland, such as the UK. In addition to exploring the influence of vaccination of inpatients and/or healthcare workers on the explored outbreaks.

Unsupervised ML (K-modes clustering) can help in predicting RTI outbreaks in acute Irish hospitals. Replacing individual diagnostic codes with diagnostic groups developed by HC led to better prediction results. Further analysis identified five diagnostic codes which contributed with two thirds of all RTIs hospitalisations, which if monitored, early warning systems can be introduced in hospitals to alert them to potential increases in hospitalisations and outbreaks as well as design vaccination programmes.

## Ethical statement

Ethics to access and analyse HIPE data was obtained from the research ethics committee of University College Dublin.

## Author contributions

Doaa Amin: Conceptualization, Methodology, Software, Validation, Formal analysis, Data Curation, Visualisation, Writing–Original Draft; Akke Vellinga: Conceptualization, Writing- Review & Editing, Supervision, Funding Acquisition.

## Funding

This work was funded by grant number RL-20200-03 from Research Leader Awards (RL) 2020, Health Research Board, Ireland and conducted as part of the SPHeRE Programme.

## Declarations of competing interest

None.

## Data Availability

Due to the sensitive nature of the HIPE dataset used in this study and restrictions from data providers, data had to remain confidential to protect the anonymity of patients and hospitals, and would not be shared.
