# Supplementary material for: Utilising unsupervised machine learning to predict outbreaks of respiratory tract infections in acute Irish hospitals (2016-2021)
Source: Public Health Pract (Oxf). 2026 Feb 7;11:100748. doi: 10.1016/j.puhip.2026.100748 (PMC12925592; doi:10.1016/j.puhip.2026.100748)
Supplement: Multimedia component 1 [file mmc1.docx]

**Appendix 1: ICD-10 codes for RTIs**

| **ICD-10 Codes** | **Description** |
| --- | --- |
| A15 | Respiratory tuberculosis, bacteriologically and histologically confirmed |
| A15.0 | Tuberculosis of lung, confirmed by sputum microscopy with or without culture |
| A15.1 | Tuberculosis of lung, confirmed by culture only |
| A15.2 | Tuberculosis of lung, confirmed histologically |
| A15.3 | Tuberculosis of lung, confirmed by unspecified means |
| A15.4 | Tuberculosis of intrathoracic lymph nodes, confirmed bacteriologically and histologically |
| A15.5 | Tuberculosis of larynx, trachea and bronchus, confirmed bacteriologically and histologically |
| A15.6 | Tuberculous pleurisy, confirmed bacteriologically and histologically |
| A15.7 | Primary respiratory tuberculosis, confirmed bacteriologically and histologically |
| A15.8 | Other respiratory tuberculosis, confirmed bacteriologically and histologically |
| A15.9 | Respiratory tuberculosis unspecified, confirmed bacteriologically and histologically |
| A16 | Respiratory tuberculosis, not confirmed bacteriologically or histologically |
| A16.0 | Tuberculosis of lung, bacteriologically and histologically negative |
| A16.1 | Tuberculosis of lung, bacteriological and histological examination not done |
| A16.2 | Tuberculosis of lung, without mention of bacteriological or histological confirmation |
| A16.3 | Tuberculosis of intrathoracic lymph nodes, without mention of bacteriological or histological confirmation |
| A16.4 | Tuberculosis of larynx, trachea and bronchus, without mention of bacteriological or histological confirmation |
| A16.5 | Tuberculous pleurisy, without mention of bacteriological or histological confirmation |
| A16.7 | Primary respiratory tuberculosis without mention of bacteriological or histological confirmation |
| A16.8 | Other respiratory tuberculosis, without mention of bacteriological or histological confirmation |
| A16.9 | Respiratory tuberculosis unspecified, without mention of bacteriological or histological confirmation |
| A18.6 | Tuberculosis of ear |
| A18.8 | Tuberculosis of other specified organs |
| A19 | Miliary Tuberculosis |
| A19.0 | Acute miliary tuberculosis of a single specified site |
| A19.1 | Acute miliary tuberculosis of multiple sites |
| A19.2 | Acute miliary tuberculosis, unspecified |
| A19.8 | Other miliary tuberculosis |
| A19.9 | Miliary tuberculosis, unspecified |
| B90 | Sequelae of tuberculosis |
| B90.9 | Sequelae of respiratory and unspecified tuberculosis |
| B95 | Streptococcus and staphylococcus as the cause of diseases classified to other chapters |
| B95.0 | Streptococcus, group A, as the cause of diseases classified to other chapters |
| B95.1 | Streptococcus, group B, as the cause of diseases classified to other chapters |
| B95.2 | Streptococcus, group D and enterococcus, as the cause of diseases classified to other chapters |
| B95.3 | Streptococcus pneumoniae as the cause of diseases classified to other chapters |
| B95.4 | Other streptococcus as the cause of diseases classified to other chapters |
| B95.41 | Streptococcus, Group C, as the cause of diseases classified to other chapters |
| B95.42 | Streptococcus, Group G, as the cause of diseases classified to other chapters |
| B95.48 | Streptococcus, other specified group, as the cause of diseases classified to other chapters |
| B95.5 | Unspecified streptococcus as the cause of diseases classified to other chapters |
| B95.6 | Staphylococcus aureus as the cause of diseases classified to other chapters |
| B95.7 | Other staphylococcus as the cause of diseases classified to other chapters |
| B95.8 | Unspecified staphylococcus as the cause of diseases classified to other chapters |
| B96 | Other bacterial agents as the cause of diseases classified to other chapters |
| B96.0 | Mycoplasma pneumoniae [M. pneumoniae] as the cause of diseases classified to other chapters |
| B96.1 | Klebsiella pneumoniae [K. pneumoniae] as the cause of diseases classified to other chapters |
| B96.3 | Haemophilus influenzae [H. influenzae] as the cause of diseases classified to other chapters |
| B96.31 | Haemophilus influenzae [H. influenzae] type B, as the cause of diseases classified to other chapters |
| B96.38 | Haemophilus influenzae [H. influenzae] other specified type, as the cause of diseases classified to other chapters |
| B96.39 | Haemophilus influenzae [H. influenzae] type not specified, as the cause of diseases classified to other chapters |
| B96.5 | Pseudomonas (aeruginosa) as the cause of diseases classified to other chapters |
| A48.1 | Legionnaires disease |
| J12 | Viral pneumonia, not elsewhere classified |
| J12.0 | Adenoviral pneumonia |
| J12.1 | Respiratory syncytial virus pneumonia |
| J12.2 | Parainfluenza virus pneumonia |
| J12.3 | Human metapneumovirus pneumonia |
| J12.8 | Other viral pneumonia |
| J12.9 | Viral pneumonia, unspecified |
| J13 | Pneumonia due to Streptococcus pneumoniae |
| J14 | Pneumonia due to Haemophilus influenzae |
| J15 | Bacterial pneumonia, not elsewhere classified |
| J15.0 | Pneumonia due to Klebsiella pneumoniae |
| J15.1 | Pneumonia due to Pseudomonas |
| J15.2 | Pneumonia due to staphylococcus |
| J15.3 | Pneumonia due to streptococcus, group B |
| J15.4 | Pneumonia due to other streptococci |
| J15.5 | Pneumonia due to Escherichia coli |
| J15.6 | Pneumonia due to other aerobic Gram-negative bacteria |
| J15.7 | Pneumonia due to Mycoplasma pneumoniae |
| J15.8 | Other bacterial pneumonia |
| J15.9 | Bacterial pneumonia, unspecified |
| J16 | Pneumonia due to other infectious organisms, not elsewhere classified |
| J16.0 | Chlamydial pneumonia |
| J16.8 | Pneumonia due to other specified infectious organisms |
| J17 | Pneumonia in diseases classified elsewhere |
| J17.0 | Pneumonia in bacterial diseases classified elsewhere |
| J17.1 | Pneumonia in viral diseases classified elsewhere |
| J17.2 | Pneumonia in mycoses |
| J17.3 | Pneumonia in parasitic diseases |
| J17.8 | Pneumonia in other diseases classified elsewhere |
| J18 | Pneumonia, organism unspecified |
| J18.0 | Bronchopneumonia, unspecified |
| J18.1 | Lobar pneumonia, unspecified |
| J18.2 | Hypostatic pneumonia, unspecified |
| J18.8 | Other pneumonia, organism unspecified |
| J18.9 | Pneumonia, unspecified |
| J20 | Acute bronchitis |
| J20.0 | Acute bronchitis due to Mycoplasma pneumoniae |
| J20.1 | Acute bronchitis due to Haemophilus influenzae |
| J20.2 | Acute bronchitis due to streptococcus |
| J20.3 | Acute bronchitis due to coxsackievirus |
| J20.4 | Acute bronchitis due to parainfluenza virus |
| J20.5 | Acute bronchitis due to respiratory syncytial virus |
| J20.6 | Acute bronchitis due to rhinovirus |
| J20.7 | Acute bronchitis due to echovirus |
| J20.8 | Acute bronchitis due to other specified organisms |
| J20.9 | Acute bronchitis, unspecified |
| J21 | Acute bronchiolitis |
| J21.0 | Acute bronchiolitis due to respiratory syncytial virus |
| J21.1 | Acute bronchiolitis due to human metapneumovirus |
| J21.8 | Acute bronchiolitis due to other specified organisms |
| J21.9 | Acute bronchiolitis, unspecified |
| J22 | Unspecified acute lower respiratory infection |
| J40 | Bronchitis, not specified as acute or chronic |
| J41 | Simple and mucopurulent chronic bronchitis |
| J41.0 | Simple chronic bronchitis |
| J41.1 | Mucopurulent chronic bronchitis |
| J41.8 | Mixed simple and mucopurulent chronic bronchitis |
| J42 | Unspecified chronic bronchitis |
| J44.0 | Chronic obstructive pulmonary disease with acute lower respiratory infection |
| J44.1 | Chronic obstructive pulmonary disease with acute exacerbation, unspecified |
| J85 | Abscess of lung and mediastinum |
| J85.0 | Gangrene and necrosis of lung |
| J85.1 | Abscess of lung with pneumonia |
| J85.2 | Abscess of lung without pneumonia |
| J85.3 | Abscess of mediastinum |
| J86 | Pyothorax |
| J86.0 | Pyothorax with fistula |
| J86.9 | Pyothorax without fistula |
| J00 | Acute nasopharyngitis |
| J01 | Acute sinusitis |
| J01.0 | Acute maxillary sinusitis |
| J01.1 | Acute frontal sinusitis |
| J01.2 | Acute ethmoidal sinusitis |
| J01.3 | Acute sphenoidal sinusitis |
| J01.4 | Acute pansinusitis |
| J01.8 | Other acute sinusitis |
| J01.9 | Acute sinusitis, unspecified |
| J02 | Acute pharyngitis |
| J02.0 | Streptococcal pharyngitis |
| J02.8 | Acute pharyngitis due to other specified organisms |
| J02.9 | Acute pharyngitis, unspecified |
| J03 | Acute tonsillitis |
| J03.0 | Streptococcal tonsillitis |
| J03.8 | Acute tonsillitis due to other specified organisms |
| J03.9 | Acute tonsillitis, unspecified |
| J04 | Acute laryngitis and tracheitis |
| J04.0 | Acute laryngitis |
| J04.1 | Acute tracheitis |
| J04.2 | Acute laryngotracheitis |
| J05 | Acute obstructive laryngitis (croup) |
| J05.0 | Acute epiglottitis |
| J05.1 | Acute obstructive laryngitis (croup) |
| J06 | Acute upper respiratory infections of multiple and unspecified sites |
| J06.0 | Acute laryngopharyngitis |
| J06.8 | Other acute upper respiratory infections |
| J06.9 | Acute upper respiratory infection, unspecified |
| J09 | Influenza due to identified zoonotic or pandemic influenza virus |
| J10 | Influenza due to identified seasonal influenza virus |
| J10.0 | Influenza with pneumonia, seasonal influenza virus identified |
| J10.1 | Influenza with other respiratory manifestations, seasonal influenza virus identified |
| J10.8 | Influenza with other manifestations, seasonal influenza virus identified |
| J11 | Influenza, virus not identified |
| J11.0 | Influenza with pneumonia, virus not identified |
| J11.1 | Influenza with other respiratory manifestations, virus not identified |
| J11.8 | Influenza with other manifestations, virus not identified |
| J30 | Vasomotor and allergic rhinitis |
| J30.0 | Vasomotor rhinitis |
| J30.1 | Allergic rhinitis due to pollen |
| J30.2 | Other seasonal allergic rhinitis |
| J30.3 | Other allergic rhinitis |
| J30.4 | Allergic rhinitis, unspecified |
| J31 | Chronic rhinitis, nasopharyngitis and pharyngitis |
| J31.0 | Chronic rhinitis |
| J31.1 | Chronic nasopharyngitis |
| J31.2 | Chronic pharyngitis |
| J32 | Chronic sinusitis |
| J32.0 | Chronic maxillary sinusitis |
| J32.1 | Chronic frontal sinusitis |
| J32.2 | Chronic ethmoidal sinusitis |
| J32.3 | Chronic sphenoidal sinusitis |
| J32.4 | Chronic pansinusitis |
| J32.8 | Other chronic sinusitis |
| J32.9 | Chronic sinusitis, unspecified |
| J33 | Nasal polyp |
| J33.0 | Polyp of nasal cavity |
| J33.1 | Polypoid sinus degeneration |
| J33.8 | Other polyp of sinus |
| J33.9 | Nasal polyp, unspecified |
| J34 | Other disorders of nose and nasal sinuses |
| J34.0 | Abscess, furuncle and carbuncle of nose |
| J34.1 | Cyst and mucocele of nose and nasal sinus |
| J34.2 | Deviated nasal septum |
| J34.3 | Hypertrophy of nasal turbinates |
| J34.8 | Other specified disorders of nose and nasal sinuses |
| J35 | Chronic diseases of tonsils and adenoids |
| J35.0 | Chronic tonsillitis |
| J35.1 | Hypertrophy of tonsils |
| J35.2 | Hypertrophy of adenoids |
| J35.3 | Hypertrophy of tonsils with hypertrophy of adenoids |
| J35.8 | Other chronic diseases of tonsils and adenoids |
| J35.9 | Chronic disease of tonsils and adenoids, unspecified |
| J36 | Peritonsillar abscess |
| J37 | Chronic laryngitis and laryngotracheitis |
| J37.0 | Chronic laryngitis |
| J37.1 | Chronic laryngotracheitis |
| J38.1 | Polyp of vocal cord and larynx |
| J38.2 | Nodules of vocal cords |
| J38.3 | Other diseases of vocal cords |
| J38.4 | Oedema of larynx |
| J38.5 | Laryngeal spasm |
| J38.6 | Stenosis of larynx |
| J38.7 | Other diseases of larynx |
| J39 | Other diseases of upper respiratory tract |
| J39.0 | Retropharyngeal and parapharyngeal abscess |
| J39.1 | Other abscess of pharynx |
| J39.2 | Other diseases of pharynx |
| J39.3 | Upper respiratory tract hypersensitivity reaction, site unspecified |
| J39.8 | Other specified diseases of upper respiratory tract |
| J39.9 | Disease of upper respiratory tract, unspecified |
| H66 | Ear infection |
| A48 | Other bacterial diseases, not elsewhere classified |
| A48.2 | Non-pneumonic Legionnaires' disease [Pontiac fever] |
| A48.8 | Other specified bacterial diseases |
| A49 | Bacterial infection of unspecified site |
| A49.0 | Staphylococcal infection, unspecified site |
| A49.00 | Staphylococcal infection, unspecified site |
| A49.2 | Haemophilus influenzae infection, unspecified site |
| A49.3 | Mycoplasma infection, unspecified site |
| A49.8 | Other bacterial infections of unspecified sites |
| A49.9 | Bacterial infection, unspecified |
| A70 | Chlamydial psittaci infection |
| A74 | Other diseases caused by chlamydiae |
| A74.8 | Other chlamydial diseases |
| A74.9 | Chlamydial infection, unspecified? |
| B25.0 | Cytomegalovirus pneumonitis |
| B27 | Infectious mononucleosis |
| B27.8 | Other infectious mononucleosis |
| B27.9 | Infectious mononucleosis, unspecified |
| B33.4 | Hantavirus (cardio-) pulmonary syndrome [HPS] [HCPS] |
| B33.8 | Other specified viral diseases |
| B34 | Viral infection of unspecified site |
| B34.0 | Adenovirus infection, unspecified site |
| B34.1 | Enterovirus infection, unspecified site |
| B34.8 | Other viral infections of unspecified site |
| B34.9 | Viral infection, unspecified |
| B97.0 | Adenovirus as the cause of diseases classified to other chapters |
| B97.1 | Enterovirus as the cause of diseases classified to other chapters |
| B97.4 | Respiratory syncytial virus as the cause of diseases classified to other chapters |
| B97.5 | Reovirus as the cause of diseases classified to other chapters |
| B97.8 | Other viral agents as the cause of diseases classified to other chapters |

**ICD-10 codes for COVID-19**

| **ICD-10 Codes** | **Description** |
| --- | --- |
| U07.1 | COVID-19, virus identified |
| B97.2 | Coronavirus as the cause of diseases classified to other chapters to identify the infectious agent |
| B34.2 | Coronavirus infection, unspecified site |
| U07.2 | COVID-19, virus not identified |
| U06.0 | COVID-19 ruled out |
| Z29.0 | Isolation |
| U07.3 | Personal history of COVID-19 |
| U07.4 | Post COVID-19 condition |
| U07.5 | Multisystem inflammatory syndrome associated with COVID-19 |
| U07.7 | COVID-19 vaccines causing adverse effects in therapeutic use |
| U10 | Multisystem inflammatory syndrome associated with COVID-19 |
| U10.9 | Multisystem inflammatory syndrome associated with COVID-19 |
| U08 | Personal history of COVID-19 |
| U08.9 | Personal history of COVID-19, unspecified |
| U09 | Post COVID-19 condition |
| U09.9 | post COVID-19 condition, unspecified |
| U12 | COVID19 vaccines causing adverse effects |
| U12.9 | COVID19 vaccines causing adverse effects |
| U11 | Need for immunization against COVID-19 |
| U11.9 | Need for immunization against COVID-19 |
| U04.9 | Severe acute respiratory syndrome [SARS], unspecified infection, infected (opportunistic)\|coronavirus NEC |
| O98.5 | COVID-19 in pregnancy |
| Z03.8 | Observation for other suspected diseases |
| Z11.5 | Encounter for screening for other viral diseases |
| Z51.5 | COVID-19 in palliative care |
| B97.29 | Other coronavirus as the cause of disease classified elsewhere |
| B97.21 | SARS-associated coronavirus as the cause of disease classified elsewhere |
| J12.82 | Pneumonia due to coronavirus disease 2019 |
| J12.81 | Pneumonia due to SARS-associated coronavirus |
| Z71.1 | Person with feared complaint in whom no diagnosis is made |

**Appendix 2:** Comparison between different unsupervised machine learning models

| **Prediction Model** | **Pros** | **Cons** |
| --- | --- | --- |
| K-modes clustering ^25^ | - Suitable for categorical data [1]. - Suitable for unlabelled data [2-4]. - Suitable for identifying patterns in a dataset when there is no clear outcome variable identified [2-4]. | - They are sensitive to outliers, however, this can be overcome by properly initialising the algorithm [5]. - The number of clusters “K” needed to group the data are unknown, thus a method such as Elbow has to be applied first [6]. - Longer run times are needed for large datasets and large number of clusters [7]. |
| K-means clustering [8] | - Widely implemented and easier to use [9]. - Suitable for numerical data. - Suitable for unlabelled data [2-4]. | - More efforts for data transformation/ pre-processing may be needed if data contains categorical variables [10], and for large categorical datasets, data transformation may result into data that is hard to interpret. - Similar to the K-modes clustering, the number of clusters “K” needed to group the data are unknown, thus a method such as Elbow has to be applied first [6]. |
| Hierarchical clustering [11, 12] | - Suitable for categorical as well as numerical datasets [13]. - Suitable for unlabelled data [2-4]. - Suitable for exploring hierarchical patterns in a dataset ^30^. - Doesn’t need number of clusters “K” to be defined ^30^. | - Very expensive computationally for large datasets [14]. - Sensitive to noise in the dataset [15]. |

**Appendix 3**

- **Model 1 (K = 6)**


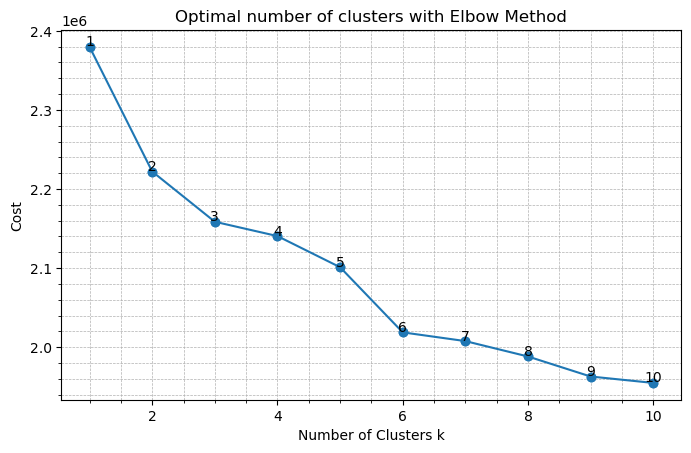


- **Model 2 (K=6)**


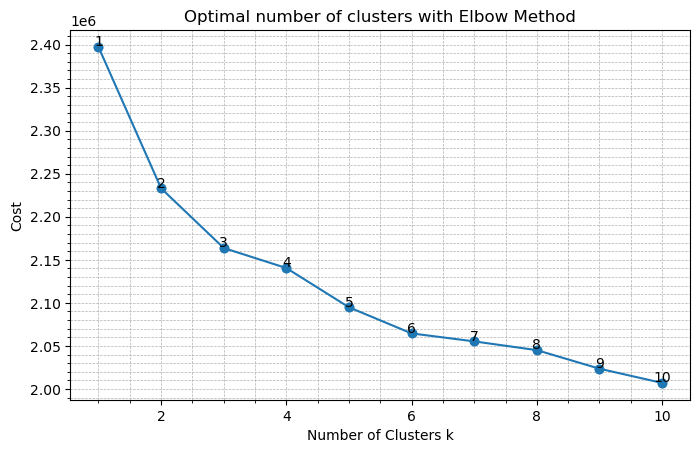


- **Model 3 (K=6)**


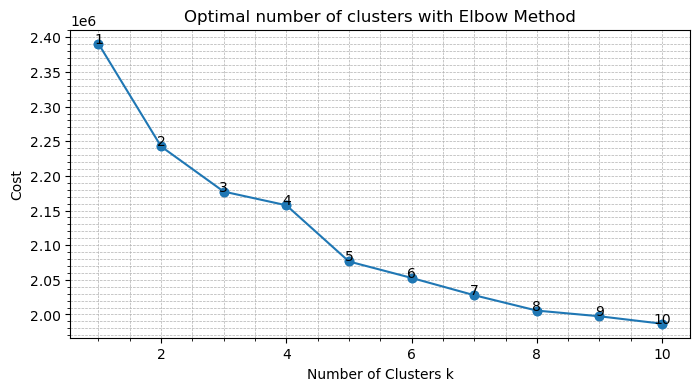


**Note:** since the K-modes clustering is based on a dissimilarity-based cost that tends to decrease in a smooth manner, the elbow can sometimes be less prominent for some datasets than others, which may explain why Model 1 showed sharper inflection point at K=6 than Models 2 and 3. In addition, any improvements after K=6 seem to be diminishing in all 3 models.

**Appendix 4**

- **Dendrogram - Model 2 - 212 Diagnostic Groups according to Hierarchical Clustering**


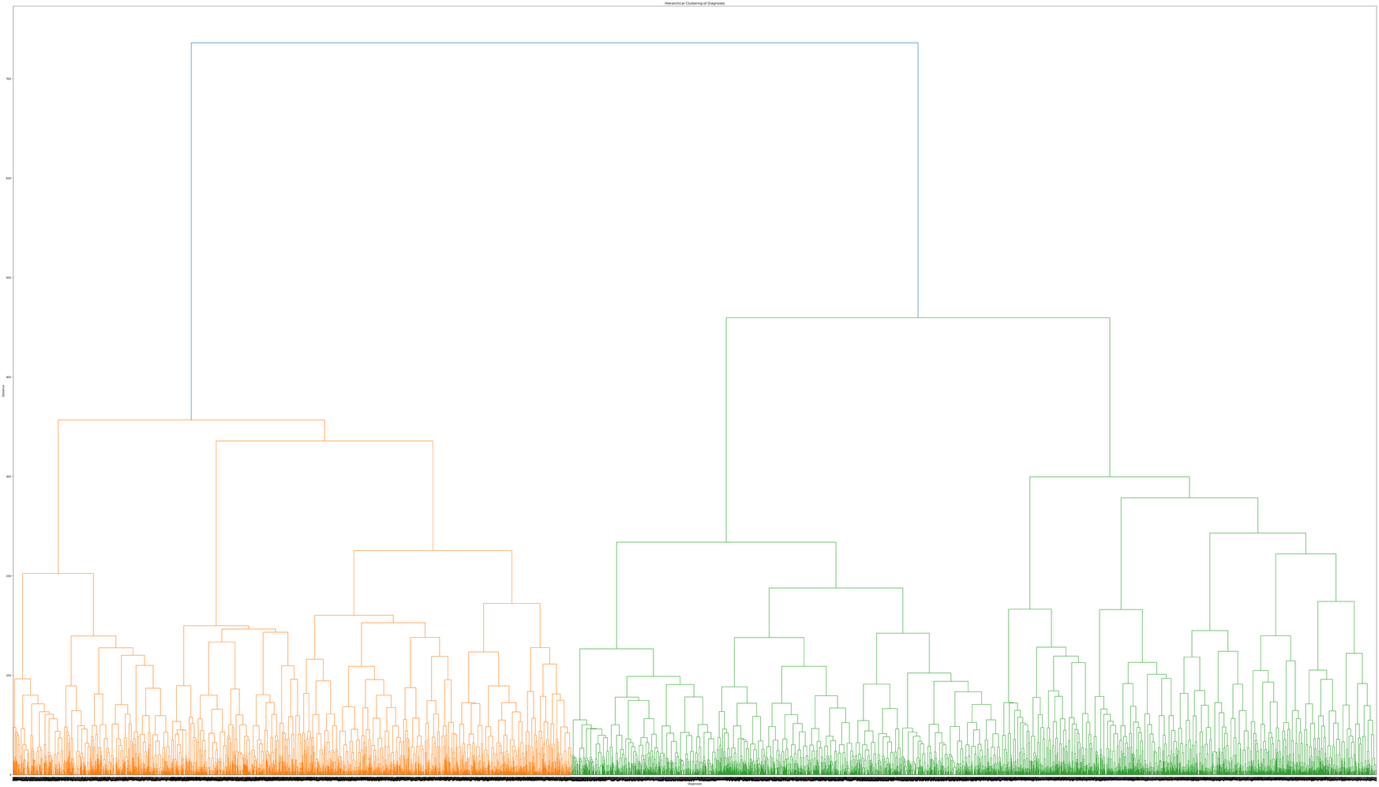


- **Dendrogram - Model 3 - 80 Diagnostic Groups according to Hierarchical Clustering**


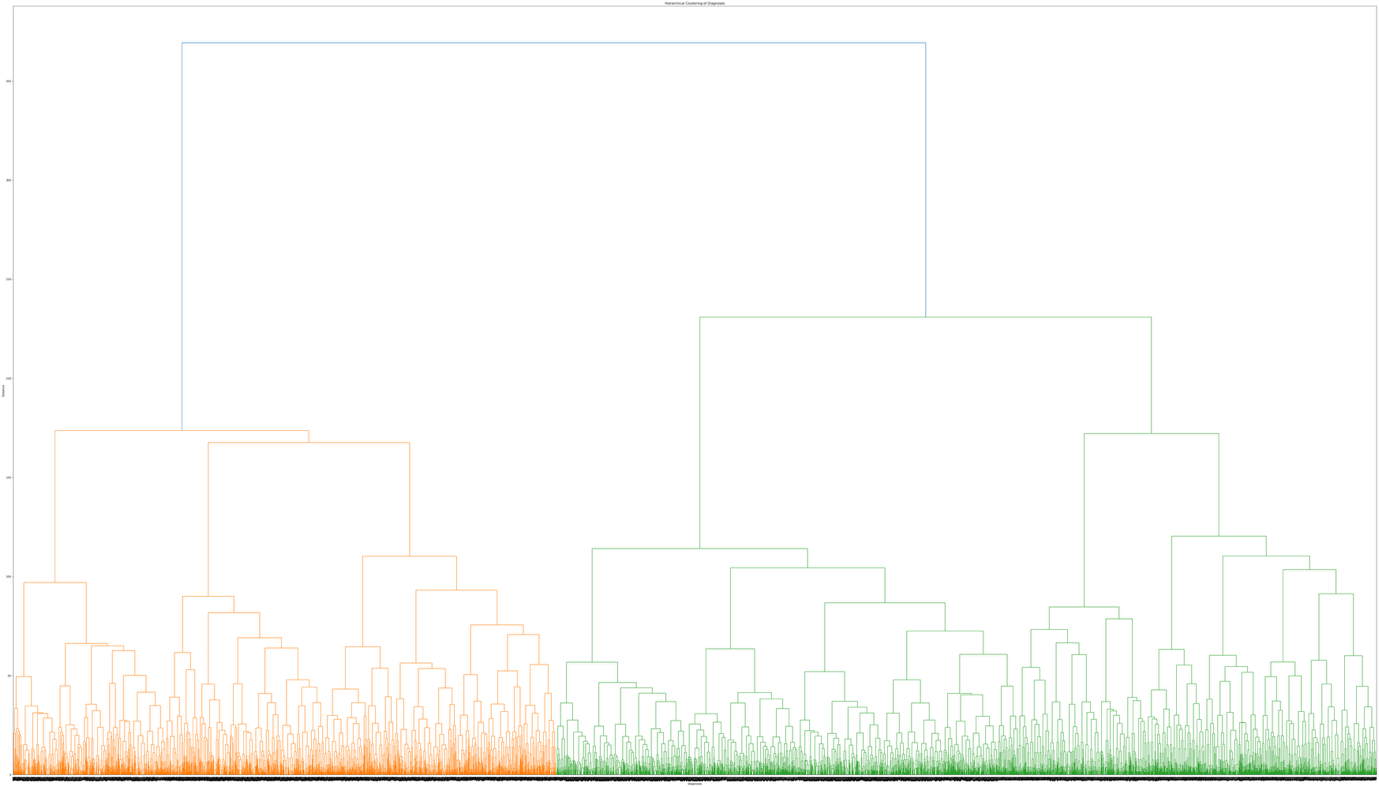


**References:**

[1] K.S. Dorman, R. Maitra. An efficient k-modes algorithm for clustering categorical datasets. Statistical Analysis and Data Mining: The ASA Data Science Journal, 15 (1) (2022). 83-97. <https://doi.org/10.1002/sam.11546>.

[2] N. Noorbakhsh-Sabet, R. Zand, Y. Zhang, et al. Artificial intelligence transforms the future of health care. The American Journal of Medicine, 132 (7) (2019). 795-801. <https://doi.org/10.1016/j.amjmed.2019.01.017>.

[3] A. Alanazi. Using machine learning for healthcare challenges and opportunities. Informatics in Medicine Unlocked, 30 (2022). 100924. <https://doi.org/10.1016/j.imu.2022.100924>.

[4] R.C. Deo. Machine learning in medicine. Circulation, 132 (20) (2015). 1920-30. <https://doi.org/10.1161/CIRCULATIONAHA.115.001593>.

[5] F. Jiang, G. Liu, J. Du, et al. Initialization of k-modes clustering using outlier detection techniques. Information Sciences, 332 (2016). 167-83. <https://doi.org/10.1016/j.ins.2015.11.005>.

[6] C. Shi, B. Wei, S. Wei, et al. A quantitative discriminant method of elbow point for the optimal number of clusters in clustering algorithm. EURASIP Journal on Wireless Communications and Networking, 2021 (1) (2021). 31. <https://doi.org/10.1186/s13638-021-01910-w>.

[7] M.K. Ng, M.J. Li, J.Z. Huang, et al. On the impact of dissimilarity measure in k-modes clustering algorithm. IEEE Transactions on Pattern Analysis and Machine Intelligence, 29 (3) (2007). 503-7. <https://doi.org/10.1109/TPAMI.2007.53>.

[8] K.P. Sinaga, M.S. Yang. Unsupervised k-means clustering algorithm. IEEE Access, 8 (2020). 80716-27. <https://doi.org/10.1109/ACCESS.2020.2988796>.

[9] M. Ahmed, R. Seraj, S.M.S. Islam. The k-means algorithm: A comprehensive survey and performance evaluation. Electronics, 9 (8) (2020). 1295. <https://doi.org/10.3390/electronics9081295>.

[10] M. Bilal, G. Ali, M.W. Iqbal, et al. Auto-prep: Efficient and automated data preprocessing pipeline. IEEE Access, 10 (2022). 107764-84. <https://doi.org/10.1109/ACCESS.2022.3198662>.

[11] M. Roux. A comparative study of divisive and agglomerative hierarchical clustering algorithms. Journal of Classification, 35 (2) (2018). 345-66. <https://doi.org/10.1007/s00357-018-9259-9>.

[12] N. Shahid. Comparison of hierarchical clustering and neural network clustering: An analysis on precision dominance. Scientific Reports, 13 (1) (2023). 5661. <https://doi.org/10.1038/s41598-023-32790-3>.

[13] D. Chen, D.-W. Cui, C.-X. Wang, et al. A rough set-based hierarchical clustering algorithm for categorical data. International Journal of Information Technology, 12 (3) (2006). 149-59.

[14] C.K. Reddy, B. Vinzamuri. A survey of partitional and hierarchical clustering algorithms. Data clustering. Chapman and Hall/CRC; 2018: 87-110.

[15] F. Ros, S. Guillaume. A hierarchical clustering algorithm and an improvement of the single linkage criterion to deal with noise. Expert Systems with Applications, 128 (2019). 96-108. <https://doi.org/10.1016/j.eswa.2019.03.031>.
